# Study on Microstructure of Fiber Laser Welding of CoCrCuFeNi High Entropy Alloy

**DOI:** 10.3390/ma15248777

**Published:** 2022-12-08

**Authors:** Juan Li, Honglong Zhao, Nian Zhou, Yingzhe Zhang, Qingdong Qin, Daoyi Wang, Jianguo Jiao, Guoli Tang, Yonghua Li

**Affiliations:** 1 Key Laboratory of Light Metal Materials Processing Technology of Guizhou Province, Guizhou Institute of Technology, Guiyang 550003, China; 2 2011 Special Functional Materials Collaborative Innovation Center of Guizhou Province, Guiyang 550003, China; 3 Guizhou Colleges and Universities Process Industry New Process Engineering Research Center, Guiyang 550003, China; 4 Guizhou Hangrui Aviation Precision Parts Manufacturing Co., Ltd., Zhunyi 563000, China

**Keywords:** high-entropy alloy, optical fiber laser welding, microstructure, mechanical properties

## Abstract

A CoCrCuFeNi high-entropy alloy was successfully welded in this study using fiber laser welding. The effects of the welding parameters on the microstructure and mechanical properties were studied. Three zones were formed: the fusion zone, partial melting zone, and base metal. The base metal exhibited a typical dendrite structure, and the Cu element segregated in the interdendrite. The fusion zone consisted of fine equiaxed crystals and columnar crystals with the same crystalline structure as the base metal. The fusion zone exhibited minimal compositional microsegregation after laser welding. Electron backscatter diffraction results showed that the low-angle grain boundary fraction in the fusion zone increased. Furthermore, some dislocations and dislocation pile-ups were present in the fusion zone, and the densities of the dislocations and dislocation pile-ups were higher than those of the base metal. The hardness of the fusion zone was considerably higher than that of the base metal, while the ultimate tensile strength and elongation values were lower than those of the base metal for all conditions. The ultimate tensile strength and the elongation increased gradually and then decreased with increasing laser power. The maximum ultimate tensile strength exceeded that of the base metal by 90%.

## 1. Introtuction

High-entropy alloys (HEAs) are a new type of alloy system. The main difference in these alloys compared with traditional alloys is that they are multi-component alloys with equimolar or near-equimolar compositions [1,2,3]. The unique compositions produce the four well-known core properties: high configurational entropy, sluggish diffusion, severe lattice distortion, and so-called “cocktail” effects [4,5]. Therefore, HEAs present unique properties, such as high thermal stability, an excellent strength/ductility combination, outstanding impact toughness, and enhanced high-temperature corrosion/oxidation/wear resistance [6]. Among the HEA systems, CoCrCuFeNi is a well-known system that has a single face-centered cubic (fcc) structure with outstanding comprehensive performance, such as high-temperature wear, irradiation stability, antibacterial ability, and so on [7,8,9].

Fusion welding is a key joining method in which part of the material is melted, enabling the fabrication of complex-shaped structures [10]. Fusion welding technologies have significant potential value for future application-oriented research and technological developments linked to the discovery of new structural materials [10], such as HEAs. Therefore, assessing the welding metallurgical process and weldability of these new materials, such as HEAs, is critical. To date, studies on CoCrCuFeNi alloys have mainly focused on their as-cast or heat-treated states, and the resulting properties [7,8,9]. The welding metallurgical process of the CoCrCuFeNi HEA has been studied scarcely. Owing to the characteristics of fusion welding, such as the existence of fast heating and cooling cycles and high peak temperatures, as well as the resulting non-equilibrium solidification [10], low-melting-point elements have a significant effect on it.

For CoCrCuFeNi HEAs, copper element can precipitate between the grains to form a low-melting-point copper-rich phase [8,9]. The higher the copper content is, the greater the volume fraction of the copper-rich phase is [11,12]. During fusion welding, the last solidified low-melting-point Cu-rich phase in the FZ produces thermal cracks under the influence of stress, resulting in welding failure. The existence of the Cu-rich phase brings difficulties to fusion welding. Past studies that have addressed the welding metallurgical process of the CoCrCuFeNi HEA primarily concentrated on pulsed laser welding [13] or dissimilar metal welding [14]. For instance, Wu et al. [13] studied the weldability by Nd: YAG pulsed laser welding and found the existence of hot cracks. Moreover, they [14] also studied the welding metallurgical process of CoCrCuFeNi and AlCoCrFeNi, and found that successful welding can be achieved. However, cracks and elemental segregation were observed in the fusion zone (FZ), which resulted in poor mechanical properties. Moreover, only one work has focused on fiber laser welding [15]. Fan et al. [15] used fiber laser welding to join the CoCrCuFeNi HEA, obtaining higher microhardness and yield stress than that of the base metal (BM). However, solidification cracks and liquation cracks were also observed. Therefore, it is necessary to carry out welding metallurgy and weldability research to solve the problems of cracks and defects.

In the present work, in order to achieve high-quality welding of the CoCrCuFeNi HEA, an IPG Photonics fiber laser was used. Considering the important influence of heat input on the generation of hot cracks, different laser powers were selected for the CoCrCuFeNi HEA welding. The relationship between laser power and microstructure change was studied. Meanwhile, the corresponding relationship between microstructure and mechanical properties was studied.

## 2. Materials and Experiments

The equal-atomic-ratio CoCrCuFeNi HEA was fabricated from Co (particle size: 125 μm), Cr (particle size: 150 μm), Cu (particle size: 50 μm), Fe (particle size: 50 μm), and Ni (particle size: 75 μm) metal powders with a purity of 99.95% with a CXZGX-1 vacuum suspension induction furnace with casting into a graphite cavity mold (size: 200 × 100 × 3 mm^3^). The equal-atomic-ratio powder (Co, Cr, Cu, Fe, and Ni) blends were dry-mixed for 8 h in a cylindrical stainless-steel jar by mechanical rotation at 100 rpm, and then were uniaxially pressed into cylindrical compacts with sizes of 20 mm diameter and 20 mm length under pressure of 100 MPa. After being dried in an oven at around 110 °C for 1 h to remove any trace of moisture, approximately 15 compacts were melted in the vacuum suspension induction furnace at 1500 °C for 10 min under argon conditions in a water-cooled copper crucible. Then, the melt was poured into a graphite cavity mold to produce the CoCrCuFeNi HEA ingot. The ingot was cut into cubes with the size of 20 × 20 × 20 mm^3^ and then remelted again. All ingots were remelted 5 times to ensure homogeneity and the top parts of the ingots containing defects were removed. The HEA was cut into small sheets of area 20 mm × 20 mm with a thickness of 3 mm with the use of an electric spark cutting for laser welding.

The samples were ground with 2500# (~6.5 μm) silicon carbide abrasive paper and then ultrasonically cleaned in alcohol before welding. The prepared samples were assembled into a butt joint using a clamping apparatus. The weld was performed using an IPG Photonics (Beijing, China) fiber laser (YLS-10000). According to previous research results, a speed of 1 m/min and an Ar flow of 25 L/min were used. Laser powers of 1.65, 1.75, 1.85, and 1.95 kW were selected to study the effect of power on microstructure and properties. The welding experiments for each laser power were repeated at least 3 times to ensure the reliability and repeatability of the research.

Microstructures, chemical composition, and phase on the cross-sections of the joints were examined with an optical microscope (OM) (Axio Imager A2M, Zeiss, Jena Germany), a scanning electron microscope (SEM) (Nova NanoSEM 450, Thermo Fisher Scientific, Waltham, MA, USA) equipped with back-scattered electrons (BSE), an energy-dispersive spectrometer (EDS) (Oxford X-act, Oxford Instruments Inc., Bethesda, MD, USA), an electron backscatter diffraction (EBSD) device (NordlysMax2, Oxford Instruments Inc., Bethesda, MD, USA), an electron probe X-ray micro-analyzer (EPMA) (JXA-8530F PLUS, accelerating voltage: 15 kV, beam current: 50 nA), an X-ray diffraction (XRD, Malvern Panalytical Ltd, Malvern, UK) device (SmartLab 9X, scanning rate: 1°/min), and a transmission electron microscope (TEM) (TECNAI G2 F20, FEI Company, Hillsboro, OR, USA). The metallographic samples for OM and SEM were etched with an aqua reagent solution (HCl: HNO_3_ of 3:1) for approximately 10 s. The samples for EBSD were polished using an Arion beam system for 2 h without solution etching. The samples for XRD were cut into a series of sheets of 20 × 20 × 5 mm^3^. The surfaces were polished with 2500# (~6.5 μm) silicon carbide abrasive paper. The samples for TEM were cut into 0.3 mm thin sheets with a precision cutting machine (MINITOM, Struers, Beijing, China), and then ground to 100~150 μm with abrasive paper. The ground sheets were punched into discs with a diameter of 3 mm. The discs were further thinned to form holes in thin areas by using an electrochemical polishing machine (Struers Tenupol-5, Struers, Ballerup, Denmark). Electrochemical thinning was carried out with an alcohol–perchloric acid (95:5) mixed solution as the electrolyte at −25 ℃ and 20 V voltage.

**Figure 1:**
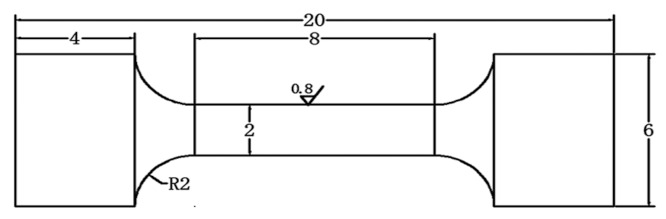
Dimensions and schematic of the tensile specimens (thickness: 3 mm).

The Vickers microhardness of the welded joints was measured according to ISO 6507-4 using a micro-hardness tester (Buehler VH3100C (Buehler, Lake Bluff, IL, USA), load: 100 g, indentation time: 10 s). All the samples were measured through a 45 × 24 matrix under the same conditions (45 points in the horizontal direction and 24 points in the vertical direction, with indentations at 0.1 mm intervals), and micro-hardness distribution maps were drawn. According to GB/T228 (Chinese standard), tensile specimens with a width of 6 mm and a length of 20 mm were machined with the electric spark cutting machine. A sketch of a tensile sample is shown in Figure 1. Subsequently, the specimens were ground with 2500# silicon carbide to ensure a smooth surface. The tensile strength tests were performed using an MTS material machine (MTS Logistics, Izmir, Turkey) (SHT5305) with a speed of 1 mm.min^−1^.

## 3. Results and Discussion

### 3.1. Basic Characteristics of Base Material

**Figure 2:**
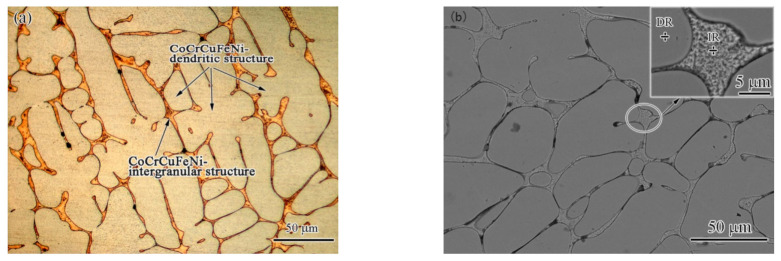
(**a**) OM and (**b**) SEM (backscattered electron) photograph of base metal.

It is well known that CoCrCuFeNi HEA is mainly composed of an fcc-structured solid solution (sometimes containing a few copper-rich phase peaks in XRD) alloy with five equal-atomic-ratio elements [16]. It was reported that the exact crystal structure of these alloys includes two phases: a high-entropy fcc solid solution phase and a (Cu) solid solution phase [17]. The high-entropy fcc phase is characterized by coarse and well-developed dendrites, and the (Cu) phase crystallizes in the interspacing of the high-entropy fcc phase [17]. In this work, the microstructure of the as-cast HEA base material was similar to that in the literature [17], and it was also a typical dendritic (DR) structure, as shown in Figure 2. The average width of the dendritic arms was ~20 μm. The DR was the high-entropy fcc phase, according to the previous studies [18] and the literature [17]. The EDS results of the DR (Table 1) show that the high-entropy fcc solid solution phase was mainly composed of Co (23.49 at.%), Cr (23.14 at.%), Fe (23.49 at.%), Ni (19.44 at.%), and a small amount of Cu (9.32 at.%). Meanwhile, the intergranular (IR) part was a Cu-rich solid solution phase, according to the EDS results in Table 1. Its composition was 79.42 at.% of Cu and microscale amounts of other elements, namely Co (3.71 at.%), Cr (4.17 at.%), Fe (4.38 at.%), and Ni (8.32 at.%). Due to the presence of the low-melting-point copper-rich phase, the CoCrCuFeNi welded joints were sensitive to hot cracking, which made the welding difficult [19]. The copper segregation phenomenon was explained by Campo et al. [11] and Zhang et al. [13], who considered the positive mixing enthalpy of copper–nickel, copper–iron, copper–chromium, and copper–cobalt, which resulted in the low solubility of copper in the HEA. The Cu-rich phase has a direct impact on the processability of the material. The question of how to eliminate the influence of copper is a key issue in the welding of this type of alloy.

### 3.2. Weld Structure and Microstructure

**Figure 3:**
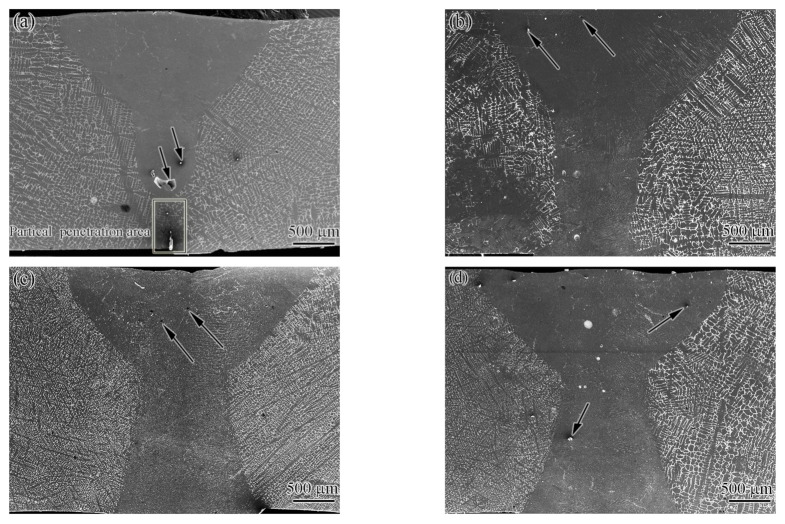
Macroscopic cross-section of SEM (secondary electron) images of the welded joint: (**a**) 1.65 kW, (**b**) 1.75 kW, (**c**) 1.85 kW, and (**d**) 1.95 kW.

Usually, the laser-welded joint of the CoCrCuFeNi HEA consisted of three zones: the FZ, partial melting zone, and BM. The heat-affected zone was narrow, and its grain size was similar to that of the BM, as reported by Verma et al. [8]. Figure 3a–d show macroscopic cross-sections of the SEM (secondary electron) images of the joints achieved at 1.65, 1.75, 1.85, and 1.95 kW, respectively. No cracks were found in any joint. However, when the laser power input was 1.65 kW, the joint did not penetrate completely, forming a 500-μm-thick incomplete penetration area (black rectangular frame in Figure 3a). When the power increased to no less than 1.75 kW, the joints were all fully penetrated and the FZ resembled an hourglass. The joints obtained at each power value all had a small number of pores (black arrows in Figure 3a–d). Undoubtedly, these defects will be reflected in the performance. It is well known that the beam power in the laser welding process plays an important role in the improvement of the geometry and morphology of the joint [20,21]. The simulated results indicated that with the increase in laser peak power, the melt pool penetration and width of the FZ gradually increased in the laser welding of Ti6Al4V alloy [21]. This result is consistent with the present study.

**Figure 4:**
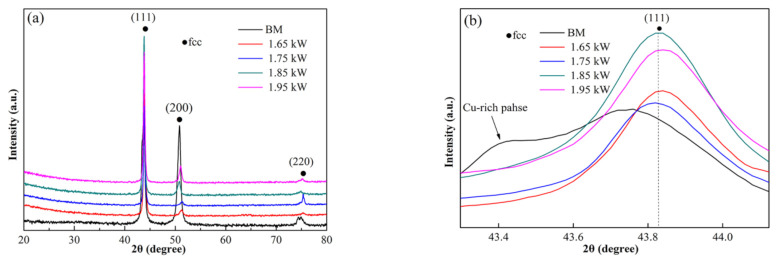
XRD patterns of the FZ at different laser powers: (**a**) XRD patterns and (**b**) detailed scans for the (111) peak.

Figure 4 shows the XRD patterns of the BM and FZs of different laser powers. It indicates that the BM was composed of an fcc structure and a very small amount of Cu-rich phase peaks (marked by an arrow in Figure 4b), which is consistent with the EDS results in Table 1. However, the FZ was composed of a single fcc solid solution phase, as shown in the detailed scans for the (111) peak (Figure 4b). The results show that the segregation of Cu was reduced in the FZ. According to the EDS results in Table 1, the composition of IR was essentially balanced, but Cu was slightly higher—that is, the atomic percentages of Co, Cr, Cu, Fe, and Ni were 19.31, 19.07, 22.24, 19.71, and 19.67 at.%, respectively. Similarly, the solid solution of Cu in DR also had a significant increase. The content of Cu increased from 9 at.% in the BM to 14 at.% in the FZ, as indicated in Table 1. It can also be seen from Figure 4b that the peaks shifted to a higher angle for all the welded samples, suggesting a decrease in the lattice parameter. It was calculated from the XRD results that the lattice parameter changed from 0.3592 nm in the BM to 0.3524 nm in the FZ. The increase in solid solubility resulted in a decrease in lattice parameter during the laser welding process [15]. Generally, an increase in solid solubility and lattice distortion will lead to an improvement in hardness and strength [22]. According to the phase structure, the joints’ performance will not be reduced significantly.

**Figure 5:**
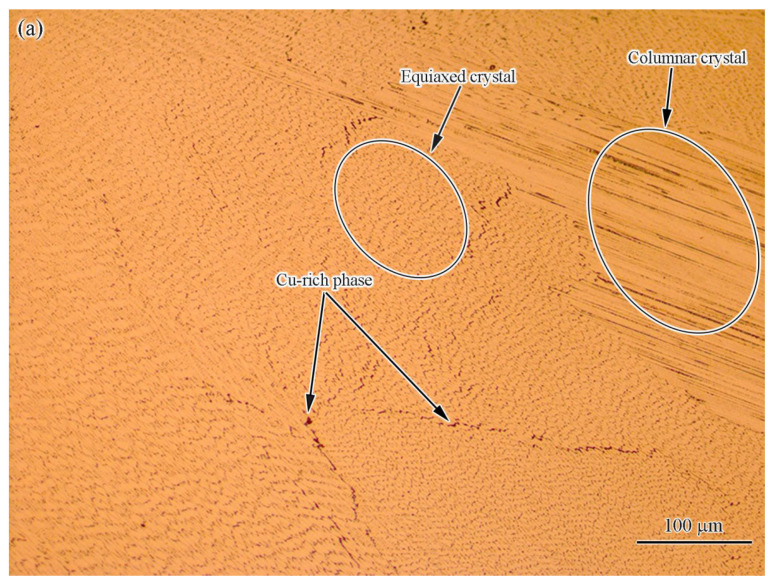

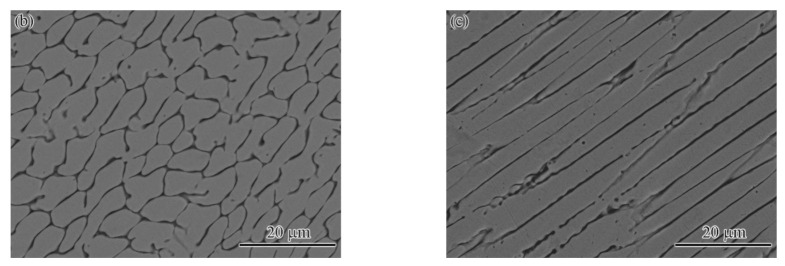
Microstructure of the FZ center of the welded joint at a laser power of 1.85 kW: (**a**) OM, SEM (backscattered electron) images of the (**b**) equiaxed crystals and (**c**) columnar crystals.

As the microstructures at different laser powers exhibited similar characteristics, the welded joint of 1.85 kW was studied as a typical example. Figure 5a shows the microstructure at the center of the FZ, which was characterized by some directionally grown columnar crystals and equiaxed crystals. These columnar crystals and equiaxed crystals were separated by the Cu-rich phase (its composition was close to that of the FZ in Table 1). The exact characteristics of the equiaxed crystals and columnar crystals are shown in Figure 5b,c, where Figure 5b shows equiaxed crystals and Figure 5c shows columnar crystals. This shows that the size of the equiaxed crystal was ~10 μm, and the size of columnar crystal exceeded 600 μm. According to the EDS result, the compositions of the equiaxed and columnar crystals were also of the high-entropy fcc solid solution phase, and the element composition and atomic percentage were similar to the data in Table 1 (FZ: DR phase). The composition and element atom percentage of the intergranular phase were consistent with the IR above, as shown in Table 1 (FZ: IR phase).

**Figure 6:**
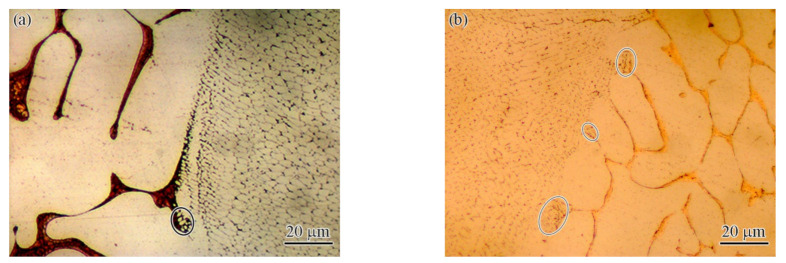

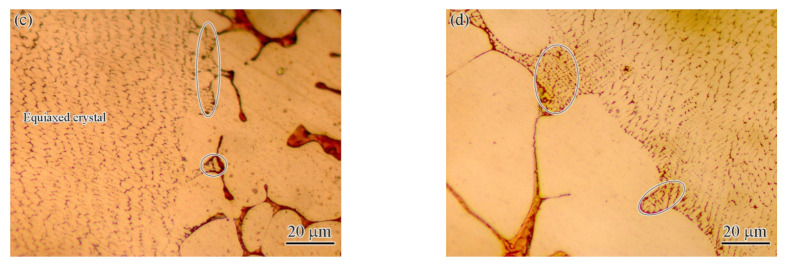
OM microstructure of the fusion line with different input powers: (**a**) 1.65 kW, (**b**) 1.75 kW, (**c**) 1.85 kW, and (**d**) 1.95 kW.

**Figure 7:**
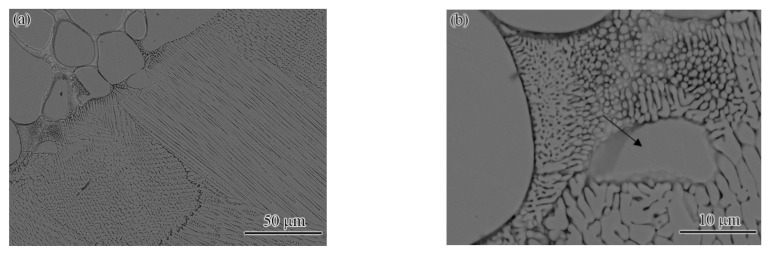
The SEM (backscattered electron) images of exact morphologies of the fusion line: (**a**) low magnification and (**b**) high magnification.

For welding, the fusion line area is another zone with weak mechanical properties. To study the influence of laser power on the fusion line, the fusion line zones of the joints obtained with different laser powers are presented in Figure 6 (marked with the black oval box). It is shown that a small amount of fine crystals was generated in the fusion line region. The exact morphology of the generated fine crystals is shown in Figure 7. The fine crystals were present as spherical or fibrous shapes with a size of ~1 μm. It can be inferred that the fusion line region is a combination of melting and partial melting. Firstly, the low-melting-point Cu-rich phase in the fusion line region towards the weld pool is completely melted, while the phase towards the BM is partially melted. Secondly, the high-melting-point fcc structure with the high-entropy phase presents partial melting in the region. Therefore, the residue of high-entropy phase melting was formed, as in in Figure 7b (marked with black arrow). The lower right side of the residual phase is the equiaxed crystal formed after complete melting and solidification, and the upper left side is the spherical phase formed after partial remelting and rapid solidification. To a certain extent, the fine spherical phase has a pinning effect, which has a positive influence on the improvement in performance. The pinning effect usually appears in the field of nanomaterials and coating materials, e.g., the fracture strength and toughness of nanocomposites are synchronously improved by the pinning effect [23]. It is worth noting that, except for the change in the content of each element, the composition of the grain boundary and the intragranular part does not change, according to the literature [16,24] and the present study.

**Figure 8:**
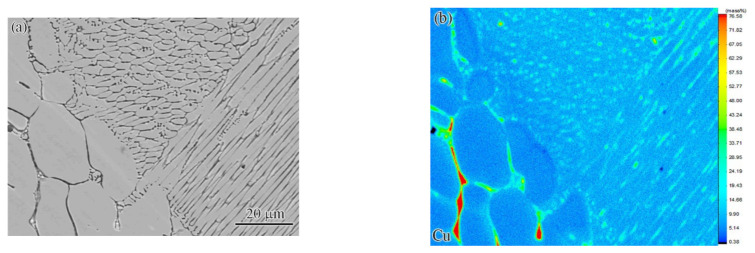
EMPA compositional mapping of Cu at a laser power of 1.85 kW: (**a**) micro-morphology and (**b**) element distribution of Cu.

In order to study the element redistribution in the fusion line region, an EPMA analysis (Figure 8) was conducted. The result indicated that the segregation of Cu in the FZ was obviously milder than that of the BM. The fine grain structure in the FZ increased the area of the IR, which had a positive role in reducing the micro-segregation of Cu. The phenomenon of segregation decrease in FZ was already reported by Wu et al. [25] for the electron beam welding of CoCrFeMnNi. It may be attributed to the large temperature gradients, rapid cooling, and the evaporation of Cu.

### 3.3. Misorientation Angle Analysis and Texture

**Figure 9:**
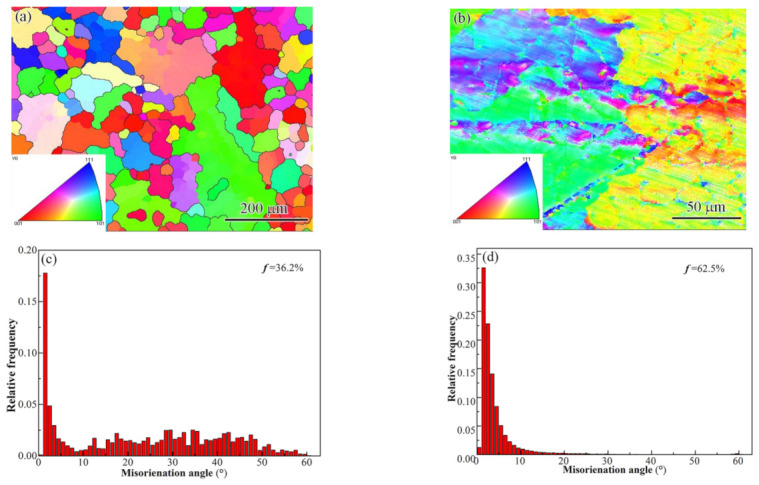
EBSD images (grain boundary + IPF) of the CoCrCuFeNi HEA: (**a**) the base metal and (**b**) fusion zone; relative frequency of misorientation angles: (**c**) the base metal and (**d**) fusion zone.

The crystal structure change was studied by EBSD. The inverse pole figure (IPF) and misorientation angles are shown in Figure 9. The figure shows that the grain size was smaller in the FZ region (Figure 9a,b), which is consistent with the OM microstructures. The change in grain size is mainly caused by the increase in cooling rate. This is also the difference between laser welding and pressure processing. In pressure processing, recovery and recrystallization are the main reasons for grain refinement, as studied by Haghdadi et al. [6,26]. Figure 9c,d show that the fractions of high-angle grain boundaries and low-angle grain boundaries in the BM and FZ are significantly different. The low-angle grain boundary fractions are 36.2% (in the BM) and 62.5% (in the FZ), respectively. This indicates a remarkable increase in the low-angle grain boundaries in the FZ. This change in grain boundary type represents a possible change in dislocation density. TEM analysis confirms this phenomenon. A similar transformation was also found in another HEA friction stir welding process [27]. The increase in dislocations will improve the hardness and strength of the material.

**Figure 10:**
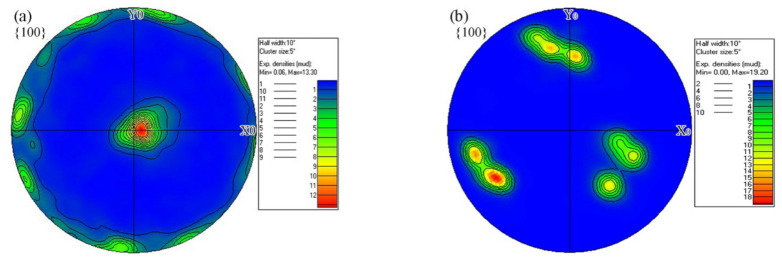
(**a**) Base metal and (**b**) fusion zone pole figures of the {001} planes.

Figure 10 presents the {100} pole figures of the BM and FZ. It shows that the orientations of the BM were mainly concentrated on (001) and (110) and that of the FZ centered at (−1 −1 0), (1 −1 0), and (011). The maximum pole density increased from 13.3 in the BM to 19.2 in the FZ, indicating a stronger texture, which was attributed to the directional solidification in the FZ. The directionally grown columnar crystals will cause anisotropy in the mechanical properties.

### 3.4. Analysis of Precipitated Phases and Dislocations

**Figure 11:**
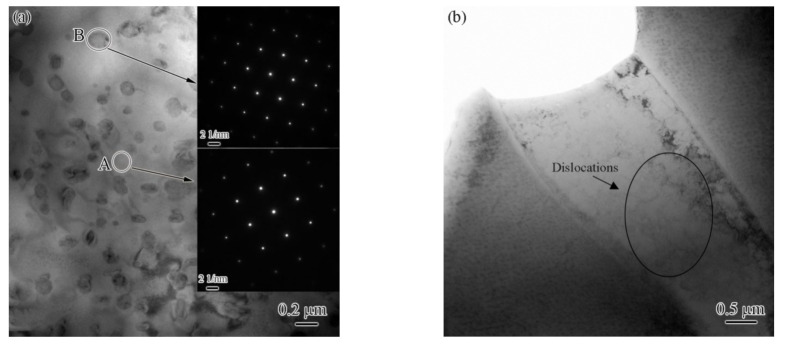

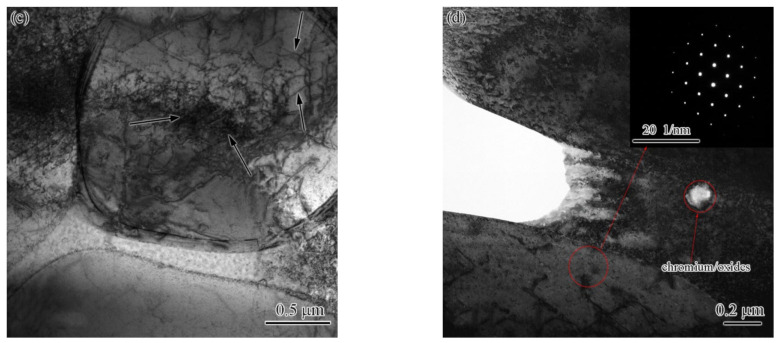
TEM images of (**a**,**b**) the BM and (**c**,**d**) the FZ.

Generally, the precipitation and dislocations of the FZ areas will change due to re-melting and rapid cooling during the welding process. Figure 11 shows TEM images with the laser power of 1.85 kW, wherein Figure 11a–b represent the BM, and Figure 11c–d represent the FZ. From Figure 11a, it can be seen that there is some granular precipitation in the matrix. The selected area’s electron diffraction results show that both the precipitation and the matrix phase present an fcc structure. In addition, the dislocation density is relatively low in the BM, as shown in Figure 11b. In contrast, the dislocation density and dislocation pile-ups increase significantly in the FZ (Figure 11c,d, black arrows). The increase in dislocation density can be associated with the high stresses owing to the rapid cooling in the FZ [28].

### 3.5. Mechanical Performance Analysis

**Figure 12:**
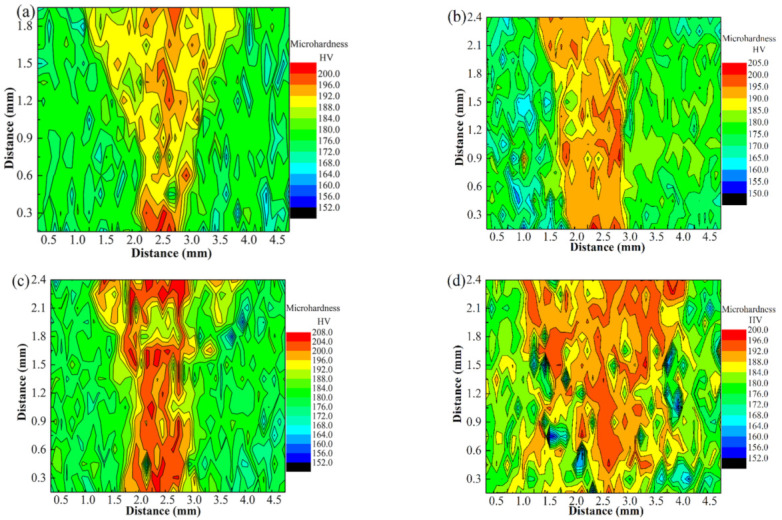
The microhardness distribution maps of the CoCrCuFeNi HEA welded joint, in which (**a**–**d**) correspond to samples 1.65–1.95 kW, respectively.

The mechanical performance of the BM and welded joints was studied in this work. Figure 12 shows the microhardness distribution maps of joints obtained under different laser powers. The distribution characteristics of the hardness values are similar to the macroscopic morphologies of the joints (Figure 3). The maps indicate higher microhardness in the FZ compared to the BM. The hardness of the joints is directly affected by the grains, dislocations, and precipitates.

The relationship between the mean grain sizes and hardness can be described by the Hall–Petch formula [14,29]:

(1) $$H=H_{0}+K_{H}d^{-\frac{1}{2}}$$

(*H*—hardness, *d*—mean grain size, *H*_0_—intrinsic hardness, *K_H_*—Hall–Petch coefficient). Thus, a decrease in grain size will cause an increase in hardness. Grains in the FZs became very fine under the rapid solidification conditions according to the microstructure analysis results, which is one of the reasons for the hardness improvement. The second factor is the dislocations and precipitated phases. As shown in the TEM images, the increase in dislocation density and the precipitation were other factors associated with the increase in hardness. Additionally, the hardness of FZ was slightly non-uniform, which may have been caused by the composition fluctuation between the columnar and equiaxed crystals. Cantor et al. [1] and Jo et al. [30] found that the local change in composition between the IR and DR regions corresponded to a 3–4% change in hardness. It is possible that the hardness fluctuation observed in the FZ was due to the local change in composition.

**Figure 13:**
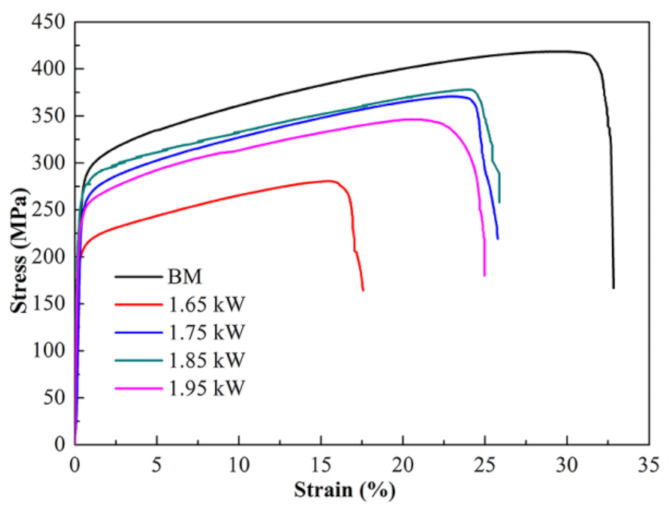
Stress–strain curve of the BM and welded joints.

Figure 13 shows the stress–strain curves of the BM and the joints achieved at different laser powers. The joint welded at 1.65 kW has a UTS of 278 MPa, with an elongation of 15.8%, while the BM is 415 MPa, with an elongation of 31.5%. The UTS gradually increases from 278 MPa to 377 MPa with the laser power increased to 1.85 kW, and it then decreases to 340 MPa when the power further increases to 1.95 kW. This shows that excessive energy input may not have a positive impact on the mechanical properties, in addition to overheating the melt. Similar results were obtained for the fiber laser welding of a CoCrFeMnNi HEA [31] and QP980/press-hardened 22MnB5 steel [32]. The mechanical properties of the joints were lower than those of the base metal, but exceeded 90%. The increasing dislocation density and the precipitated phase reduced the plasticity of the welded joints. Although the strength exceeded that of the BM by 90%, it was still lower. One possible reason for the strength loss is the formation of inclusions and residual stress. According to the SEM and TEM results, there were some defects and inclusions in the microstructure. With the laser power further increased to 1.95 kW, in addition to the larger grain size, the overheated melt caused an increase in the inclusions and defects.

## 4. Conclusions

The CoCrCuFeNi high-entropy alloy was successfully welded using fiber laser welding. The effects of the welding parameters on the microstructure and mechanical properties were studied. The following conclusions are thereby drawn.

(1) Three zones were formed in the welded joint: base metal, fusion zone, and partial melting zone.
(2) The base metal exhibited a typical dendrite structure composed of the CoCrCuFeNi solid solution phase, and the Cu element segregated in the interdendrite, forming a Cu-rich phase. The content of Cu in the Cu-rich phase reached 79%.
(3) The fusion zone was also composed of the CoCrCuFeNi solid solution phase and Cu-rich phase. The difference is that the solid solution phase consisted of fine equiaxed crystals (~10 μm) and columnar crystals. The Cu content of the copper-rich phase decreased significantly from 79.42 at.% to 22.24 at.%.
(4) The partial melting zone (fusion line region) generated fine crystals present as spherical or fibrous shapes with a size of ~1 μm.
(5) The low-angle grain boundary fraction was significantly increased from 36.2% in the BM to 62.5% in the FZ. High-density dislocations and dislocation pile-ups were produced at the same time.
(6) Compared with the base metal, the hardness of FZ showed a significant increase. The UTS and elongation increased first and then decreased as the laser power was increased. The maximum value of the UTS exceeded that of the BM by 90%.

The results showed that proper welding parameters can reduce the segregation of Cu obtaining better weld quality without cracks.

## Figures and Tables

**Table 1:** Composition of base metal and fusion zone (at.%).

		Co	Cr	Cu	Fe	Ni
Base metal	DR	23.49	23.14	9.32	24.61	19.44
	IR	3.71	4.17	79.42	4.38	8.32
Fusion zone	DR	21.47	21.72	14.81	21.24	19.76
	IR	19.31	19.07	22.24	19.71	19.67
